# TrkA-cholinergic signaling modulates fear encoding and extinction learning in PTSD-like behavior

**DOI:** 10.1038/s41398-022-01869-2

**Published:** 2022-03-17

**Authors:** Sudhirkumar Yanpallewar, Francesco Tomassoni-Ardori, Mary Ellen Palko, Zhenyi Hong, Erkan Kiris, Jodi Becker, Gianluca Fulgenzi, Lino Tessarollo

**Affiliations:** 1 Neural Development Section, Mouse Cancer Genetics Program, Center for Cancer Research, National Cancer Institute, Frederick, MD 21702 USA; 2 Present Address: Department of Biological Sciences, Middle East Technical University, Ankara, Turkey; 3 Present Address: Department of Molecular and Clinical Sciences, Marche Polytechnic University, Ancona, 60020 Italy

**Keywords:** Pharmacology, Psychiatric disorders, Learning and memory

## Abstract

Recent studies have suggested that the use of cognitive enhancers as adjuncts to exposure-based therapy in individuals suffering from post-traumatic stress disorder (PTSD) may be beneficial. Brain cholinergic signaling through basal forebrain projections to the hippocampus is an established pathway mediating fear response and cognitive flexibility. Here we employed a genetic strategy to enhance cholinergic activity through increased signaling of the NGF receptor TrkA. This strategy leads to increased levels of the marker of cholinergic activation, acetylcholine synthesizing enzyme choline acetyltransferase, in forebrain cholinergic regions and their projection areas such as the hippocampus. Mice with increased cholinergic activity do not display any neurobehavioral abnormalities except a selective attenuation of fear response and lower fear expression in extinction trials. Reduction in fear response is rescued by the GABA antagonist picrotoxin in mutant mice, and, in wild-type mice, is mimicked by the GABA agonist midazolam suggesting that GABA can modulate cholinergic functions on fear circuitries. Importantly, mutant mice also show a reduction in fear processing under stress conditions in a single prolonged stress (SPS) model of PTSD-like behavior, and augmentation of cholinergic signaling by the drug donepezil in wild-type mice promotes extinction learning in a similar SPS model of PTSD-like behavior. Donepezil is already in clinical use for the treatment of dementia suggesting a new translational application of this drug for improving exposure-based psychotherapy in PTSD patients.

## Introduction

Fear-based psychiatric conditions including phobias, obsessive-compulsive disorders, panic disorders, and post-traumatic stress disorder (PTSD) are among the most disabling, chronic, and prevalent disorders. PTSD frequently occurring as an aftermath of traumatic events such as threat of death, accidents, sexual violence, or childhood abuse is associated with significant individual suffering and societal burden. While the life-time prevalence of PTSD in the general population is estimated to be ~7%, it is dramatically increased (30%) in high-risk groups such as military personnel and victims of sexual assault [1–3]. Currently, treatment for PTSD comprises psychotherapy [cognitive behavioral therapy (CBT) or exposure therapy] and pharmacological agents such as serotonin reuptake inhibitors (SSRIs- sertraline and paroxetine) [4]. While combining psychotherapy with SSRIs has demonstrated considerable efficacy in controlling PTSD symptoms, a significant proportion of patients are resistant to therapy and, in nearly half the cases, there is relapse with return of pathological fear [5–7]. Thus, there is an urgent need for developing better treatment strategies.

The core features of PTSD include abnormal encoding of fear resulting in exaggerated fear response and inability to overcome or extinguish persistent, intrusive traumatic memories. Therefore, attenuation of traumatic memory or enhancement of fear extinction has been the focus of basic and translational research in the PTSD field [7–9]. Fear response, an adaptive behavioral change in response to threatening stimuli or traumatic event, is mediated by a variety of cortical and subcortical regions that include prefrontal cortex, hippocampus, and amygdala. Interestingly, each of these critical areas of the brain fear circuitries is interconnected with the medial septum and diagonal band of Broca basal forebrain cholinergic neurons (BFCN). Septo-hippocampal cholinergic projection neurons regulate cognitive flexibility particularly in regard to fear learning and extinction. Acetylcholine interacts with other modulators, neurotransmitters, and hormonal systems that are implicated in PTSD pathogenesis [10, 11]. Specifically, in animal models of PTSD-like behavior, fear conditioning is associated with an increase of acetylcholine release in the hippocampus [12] and muscarinic cholinergic blockade has been shown to free fear extinction from its contextual dependency [13] while nicotinic cholinergic agonist cotinine has been shown to enhance the extinction of fear memory and reduces anxiety after fear conditioning [14]. Moreover, M1 muscarinic cholinergic receptor knockout mice exhibit enhanced contextual fear acquisition [15]. These lines of evidence clearly establish a crucial role for cholinergic signaling in vivo in regulating fear response as well as extinction learning. Surprisingly, this crucial signaling pathway regulating neuroplasticity, particularly in learning-related processes is yet to be investigated for its ability to serve as an adjuvant to exposure therapy for PTSD patients.

The Nerve Growth Factor (NGF) receptors p75 and the tropomyosin-related kinase A (TrkA) are expressed in the cholinergic basal forebrain neurons where they regulate cholinergic neurotransmission. Together with NGF, they are also expressed in brain areas regulating fear response [16, 17]. While conditional deletion of p75 in cholinergic neurons results in reduced fear extinction consolidation, CNS deletion of TrkA in mouse does not seem to affect fear conditioning [18, 19]. Loss of cholinergic p75 altered fear extinction consolidation by increasing synaptic connectivity of cholinergic neurons targeting the prefrontal cortex [18]. Therefore, we wondered whether increasing TrkA trophic signaling can also regulate fear circuitries. Recently, we have generated a mouse model with increased endogenous TrkA signaling. Specifically, we have shown that deletion of a three amino acid domain (KFG) in the TrkA juxtamembrane region causes a reduction in ubiquitin-mediated degradation of the receptor leading to increased protein levels and an increased response to endogenous NGF in dorsal root ganglia neurons [20]. Here, we show that this mouse model has also increased TrkA protein levels in the brain basal forebrain cholinergic region and this increase is associated with an increase in the levels of the acetylcholine synthesizing enzyme choline acetyltransferase (ChAT). Interestingly, these mice have selective alterations in fear processing under conditions of stress suggesting that manipulations of BFCN neurotransmission could be targeted in conditions of stress disorders such as PTSD. Indeed, we found that pharmacological augmentation of cholinergic signaling using donepezil, a cognition enhancing drug that promotes brain cholinergic signaling, accelerated fear extinction in an animal model of PTSD-like behavior. Donepezil is an FDA-approved drug already in clinical use, suggesting its potential immediate use for the treatment of PTSD as an adjuvant to exposure-based therapies.

## Materials and methods

### Animals and drug treatment

The generation of TrkA-KFG mutant mice has been described elsewhere [20]. All tests were conducted on at least 3-month-old, age-matched, male mutants, or C57BL/6CR mice. Male mice were used to exclude the effects of sexual dimorphism and estrous phase on behavior. Animals were kept in the NCI vivarium in colony cages at an ambient temperature of 25 ± 2 °C and 45–55% relative humidity with 12 h light: dark cycle. The mice had free access to standard rodent pellet diet and drinking water. Donepezil (3 mg/kg i.p) was obtained from Sigma-Aldrich (USA). Picrotoxin (0.5 mg/kg i.p) and midazolam (1.25 mg/g i.p) were from Cayman Chemicals (USA). The drug dosage was based on results of pilot experiments and earlier publications [21–23]. TMT (2, 3, 5-Trimethyl-3-thiazoline) was obtained from PheroTech, Canada. All procedures were approved by NCI-Frederick ACUC committee and followed the National Institutes of Health Guidelines for animal care and use.

### Western blot analysis

After CO2 asphyxiation of animals, brains were quickly removed and dissected to isolate basal forebrain cholinergic region (BFCN, septum, and diagonal band of Broca), striatum, hippocampus, prefrontal cortex, amygdala, and cortex. Tissue samples were immediately frozen in dry ice. After homogenization in 1X RIPA lysis buffer (Millipore, USA), supernatants were collected, mixed with 2X Laemli sample buffer and denatured by boiling (100 °C for 5 min). Samples were then loaded on Bis-Tris gels with 1X Bis-Tris running buffer (Invitrogen, USA) and subjected to electrophoresis before transferring to PVDF membranes by electroblotting. Membranes were blocked for 1 h in 5% milk in 1X TBST and incubated with the specific primary antibody overnight at 4 °C. After washing, membranes were incubated with HRP-conjugated secondary antibody, washed again and exposed to Syngene Gene Snap Bio-Imaging System to capture the band intensities using the SuperSignal West extended duration substrate (Thermo Scientific, USA). Band intensity quantification was done using Gene Tools Software (Syngene Bio-Imaging). Antibodies (Supplementary Table 1) were as follows: rabbit anti-TrkA antibody (1:500, Advanced Targeting Systems), goat anti-ChAT (1:500, Millipore), anti-actin HRP (1:5000, SantaCruz), HRP-conjugated secondary antibodies (1:5000, Millipore). All details of the antibodies used in this study are reported in Supplementary Table 1.

### Behavioral analysis

The procedure for the general behavioral characterization of TrkA-KFG mutant mice is described in supplementary data.

#### Novel object recognition test

Animals were first subjected to habituation to the handling and testing environment by introducing them into the 16″ × 16″ × 12″ Plexiglass Chamber every day for 3 d (10 min/day). On day 4, for the object recognition task, each animal was placed into the chamber and exposed to two identical objects placed at the two corners of the chamber. Mice explored the objects for 10 min in the chamber. Object exploration was defined as the animal sniffing (directing the nose to the object at <2 cm), licking or touching the objects but not leaning against, turning around, or sitting/climbing on the object. Animals were then returned to the home cage. After a 1 h interval, mice were returned to the same test chamber but one of the objects was replaced with a novel object and mice were allowed to explore the objects for 5 min. The time spent exploring the familiar object (a) versus the novel object (b) was recorded with a video camera and used to calculate a discrimination ratio b/(a + b) to compare the performance of TrkA-KFG mice with WT mice [24].

#### Contextual fear conditioning and extinction

For contextual fear conditioning, on the first day, animals were introduced to an automated fear conditioning apparatus (Omnitech electronics, USA) for 3-min context exposure. The next day, mice were subjected to contextual conditioning by placing them in the same chamber and allowing exploration for 2 min followed by three unconditional stimuli (US, mild footshock: 0.5 mA intensity, 2 s duration) separated by a 60 s interval. Twenty-four hours later, mice were tested again for their contextual fear response by scoring freezing behavior over a 3 min session. For measurement of fear extinction, mice were subjected to up to 9 extinction trials at 24 h intervals consisting of a daily 3-min exposure to the context in the absence of US (foot shock). The ability to extinguish fear was measured by scoring freezing time over the course of the extinction trials [25].

#### Morris water maze

Hippocampus dependent spatial learning and memory test was conducted using a Morris water maze according to published protocols [26]. The test consisted of a white circular (diameter-120 cm) pool filled with room temperature water. White nontoxic tempura paint was added to make the submerged platform invisible. The square plexiglass platform (10 × 10 cm) was placed in one of the quadrants ~ 2 cm below the water level and the position of platform was maintained during the acquisition trials over a course of five days. Each day the mice were placed in the pool following a sequence of three random starting positions along the perimeter of the pool and allowed 60 s to locate the submerged platform. The time to reach the platform (escape latency) was recorded for each trial. If the animal was unable to locate the platform within 60 s, it was manually placed on the platform for 15 s before returning to home cage. On day 6, a probe trial was conducted in which the platform was removed from the pool and animals were allowed to swim from a novel starting position for 60 s. The time spent in the quadrant of the pool that originally contained the platform was recorded to determine if the animals remembered the platform location.

#### Single prolonged stress model

To create a model of a traumatic stressful event in mice, we employed a modified version of the rodent SPS paradigm by using a combination of physical and psychogenic stressors [27–29]. The modified SPS procedure consisted of three stages including a 2 h immobilization, 15 min forced swim, and 30 min exposure to fox odor. Animals were first immobilized in a Corning 50 ml tube for 2 h. A hole at the end of tube allowed the mice to breathe freely. After a 15 min recovery time in the home cage, they were then placed in 5-L glass cylinder (185 mm diameter) with water level at 3 L and allowed to swim for 15 min. Following a further 15-min recovery period, they were then exposed for 30 min to TMT (2, 3, 5-Trimethyl-3-thiazoline; PheroTech, Canada), a component of fox urine and feces that mimics exposure to predator (fox) odor and serves as a psychogenic stressor. For the exposure, we used a True-Flow tissue cassette (Fisher brand, USA) that contained a piece of filter paper soaked in 20 μl of TMT on two opposing corners of the cage [30]. Animals were then returned to the home cage and a week later they were subjected to contextual fear conditioning and extinction as described above. Such multi-model stress is believed to be a highly stressful event and is used to replicate the neurobehavioral abnormalities observed in PTSD patients [27–29].

### Statistical analysis

Data are presented as Mean ± SEM. Statistical analysis was done using GraphPad prism (GraphPad, USA) software. A ‘*p*’ value of <0.05 was considered as a statistically significant difference. Animals of the same genotype were randomly allocated into different groups. All data were collected and analyzed either by automated software or by investigators who were blind to the experimental conditions. Estimation of variation within and between groups was assessed by GraphPad prism’s in-built function. Experiments with two groups were subjected to Student’s ‘t’ test. The results of behavioral analysis were subjected to non-parametric statistical test. Experiments with more than two groups were subjected to ANOVA followed by suitable post-hoc analysis. The sample size of animals used for the behavioral analysis was based on similar studies in the literature and our own studies confirming the validity and reproducibility of the outcome from this type of analysis.

## Results

### Genetic deletion of the KFG domain in the TrkA gene causes an increase in CNS TrkA and ChAT levels

**Fig. 1:**
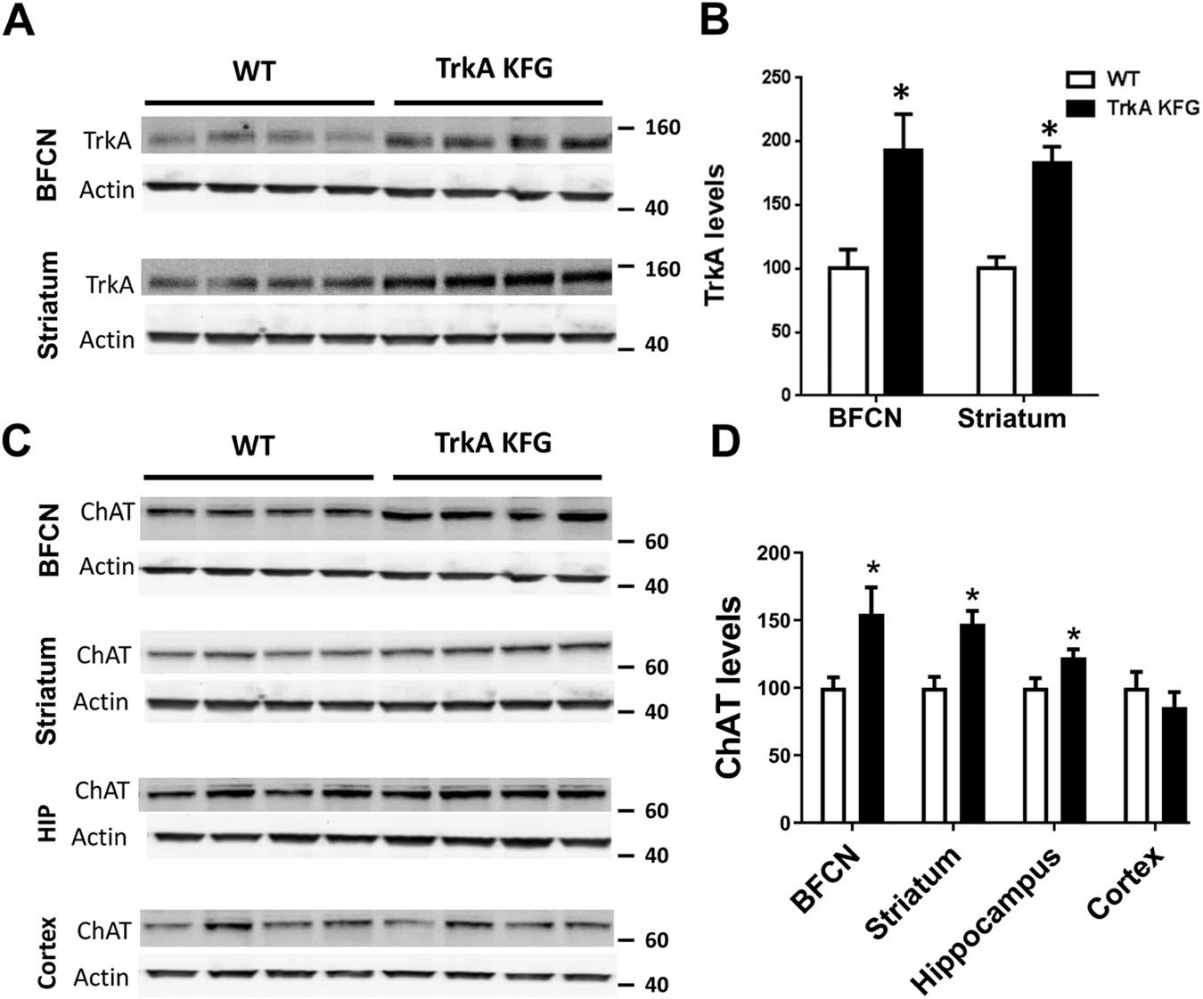
TrkA-KFG mutant mice have increased CNS levels of TrkA and ChAT. Representative western blot images showing TrkA (**A**) and ChAT (**C**) protein levels in basal forebrain cholinergic neuron (BFCN) area, striatum, hippocampus (HIP), and cortex. Molecular weight markers in KD are on the right. Note the increased TrkA protein levels in BFCN and striatum of TrkA-KFG mutant mice (**A**, **B**). Levels of ChAT protein are increased in BFCN, striatum, and hippocampus but not in cortex (**C**, **D**). Actin was used as loading control. Histograms (**B**, **D**) show results of quantitative analysis of band intensity in both genotypes. *N* = 8 per genotype; *p < 0.05 by Student’s *t* test.

TrkA is expressed in the CNS basal forebrain cholinergic neurons where, following NGF activation, it promotes cholinergic neuron development, phenotypic differentiation, and survival [24, 31]. We have previously reported that deletion of a 3 aa domain containing a ubiquitination site (KFG) in the intracellular juxtamembrane region of TrkA tyrosine kinase receptor (TrkA-KFG) leads to an increase in TrkA protein levels and signaling in isolated embryonic dorsal root ganglion (DRG) neurons [20]. Therefore, we evaluated if a similar increase in TrkA levels occurs also in the BFCN and striatum, two CNS regions expressing TrkA. Western blot analysis of BFCN and striatum showed, respectively, a 92 and 83% increase in TrkA protein levels in TrkA-KFG when compared to WT mice (Fig. 1A, B). Since we have shown previously that TrkA is not only expressed at higher levels in mutant mice but also promotes increased NGF signaling, we evaluated the levels of one of its downstream targets, choline acetyltransferase (ChAT), a marker of cholinergic function [32–34]. Western blot analysis showed an increase in the levels of ChAT in brain regions with neurons expressing TrkA including the BFCN (54% increase) and striatum (47% increase). The hippocampus, a BFCN target area also showed a small, but significant, 23% increase. However, no changes were detected in the cortex, a region where TrkA is expressed at negligible levels and is the target of the nucleus basalis of Meynert (NBM), which has few TrkA expressing neurons (Fig. 1C, D and Supplementary Fig. 1A) [35, 36]. By quantitative stereological methods we found that the number of BFCN neurons and their morphological development (cell area and volume) were comparable between genotypes suggesting that BFCN neuron postnatal development is not affected by increased TrkA levels, as seen in DRG neurons [20]. The juxtamembrane region encoding the KFG domain is conserved among all three Trk receptor genes including TrkA, TrkB, and TrkC and a number of reports have shown that TrkB and TrkC are also expressed in the BFCN. Therefore, to further test the specificity of TrkA function on BFC neuron cholinergic region we generated, by conventional gene targeting and CRISPR/Cas9 technology, mice respectively with a TrkB or TrkC receptor lacking the KFG domain (TrkB-KFG and TrkC-KFG respectively; Supplementary Fig. 2). However, basal forebrain ChAT levels in TrkB-KFG or TrkC-KFG mice were indistinguishable from those of control mice suggesting a selective role for TrkA signaling. Moreover, these results suggest that the TrkA-KFG mutant mice can serve as a cholinergic gain-of-function mouse model.

### Increased CNS TrkA levels reduce fear response

To address whether TrkA-KFG mice show any overt physical or behavioral alterations, we subjected WT and mutant mice to a comprehensive battery of physical and behavioral tests. Mutant TrkA-KFG mice showed no alterations in locomotor activity, muscle strength and coordination, anxiety- and depression-related behavior as well as behaviors involving sensory-motor functions (Supplementary Figs. 3–5).

**Fig. 2:**
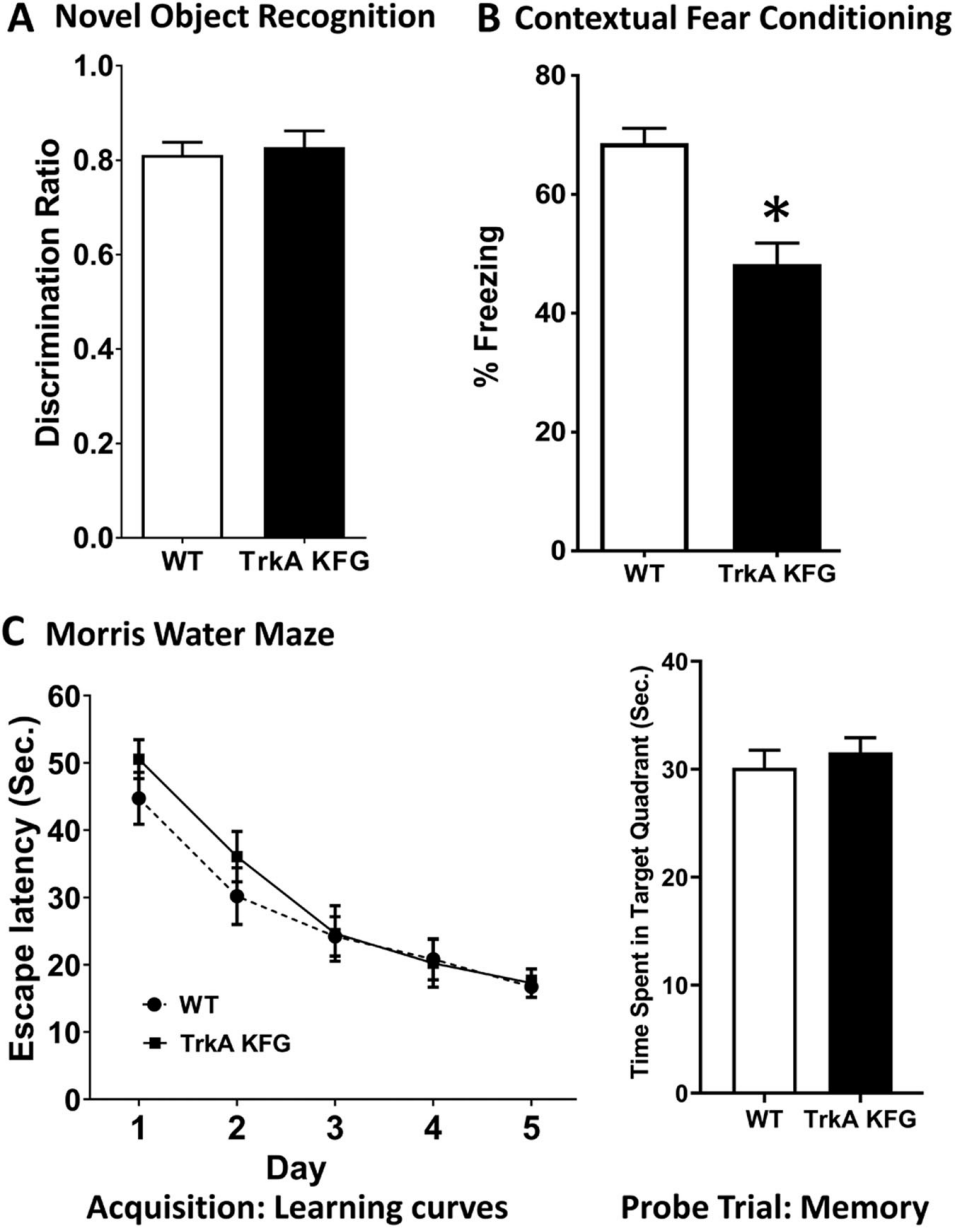
CNS TrkA upregulation selectively attenuates fear expression. TrkA-KFG mice have specific alterations in contextual fear conditioning. **A** Graphical representation of the novel object recognition test showing that WT and TrkA-KFG mice spend the same amount of time in exploring the novel versus the familiar object expressed as discrimination ratio (*n* = 8/group). **B** Graph showing that the TrkA-KFG mice have reduced freezing response in contextual fear conditioning test (% Freezing 68.63 ± 2.48 in WT and 48.26 ± 3.56 in TrkA-KFG, *n* = 8/group). **C** Results from the Morris Water maze test showing that both escape latency (left panel; time to locate the submerged platform) during the acquisition trial for spatial learning as well as the time spent in in the quadrant where the platform was previously located during the probe trial (right panel) for spatial reference memory are similar in WT (*n* = 15) and TrkA-KFG (*n* = 14) mice. Statistical analysis by Mann–Whitney test. *p < 0.05.

Next, we subjected these mice to specific behavioral tasks in which TrkA-cholinergic signaling has been implicated. We evaluated three independent learning and memory tasks including the novel object recognition test, the Morris water maze and the contextual fear conditioning test. The novel object recognition task measures attention and working memory without any added stress and relies on BFCN projections to the cortex; the Morris water maze performance depends on signaling of forebrain cholinergic projections to the hippocampus; whereas the fear conditioning test evaluates the response to a stressful event involving the septo-hippocampal circuitry [17, 24, 37–39]. When we tested WT and TrkA-KFG mice in the novel object recognition test evaluating object-in-place associative learning [24, 38, 39], we did not observe any difference in the exploration of novel versus familiar object (Fig. 2A). Similarly, in the Morris water maze, TrkA-KFG mice performed as WT controls in the acquisition and probe trial indicating that spatial learning and memory is not affected by increased TrkA levels (Fig. 2C). Interestingly, in the contextual fear conditioning paradigm, we found that, freezing response to context is selectively attenuated in TrkA-KFG mice (30% decrease, Fig. 2B). The fact that TrkA-KFG mice have attenuated fear response after conditioning without any change in the novel object recognition or Morris water tests indicates a selective alteration in fear processing.

In addition to the hippocampus, basal forebrain cholinergic projections to the amygdala and prefrontal cortex (PFC) are also important components of the fear circuitry in the brain [35, 38]. Therefore, we tested ChAT levels in these regions. Western blot analysis of dissected amygdala and PFC tissues found no differences in ChAT content between TrkA KFG and WT mice. For the amygdala this was not surprising because there are few neurons expressing TrkA in the NBM, the main BFCN region projecting to the amygdala (Supplementary Fig. 6A, B). Importantly, in agreement with the biochemical data, the performance of TrkA KFG mice in the amygdala-dependent fear response to auditory cue, was similar to that of WT mice (Supplementary Fig. 6C). Taken together, these data further suggest that a selective alteration in hippocampal TrkA-Cholinergic signaling is responsible for the reduced contextual fear response as compared to control mice.

**Fig. 3:**
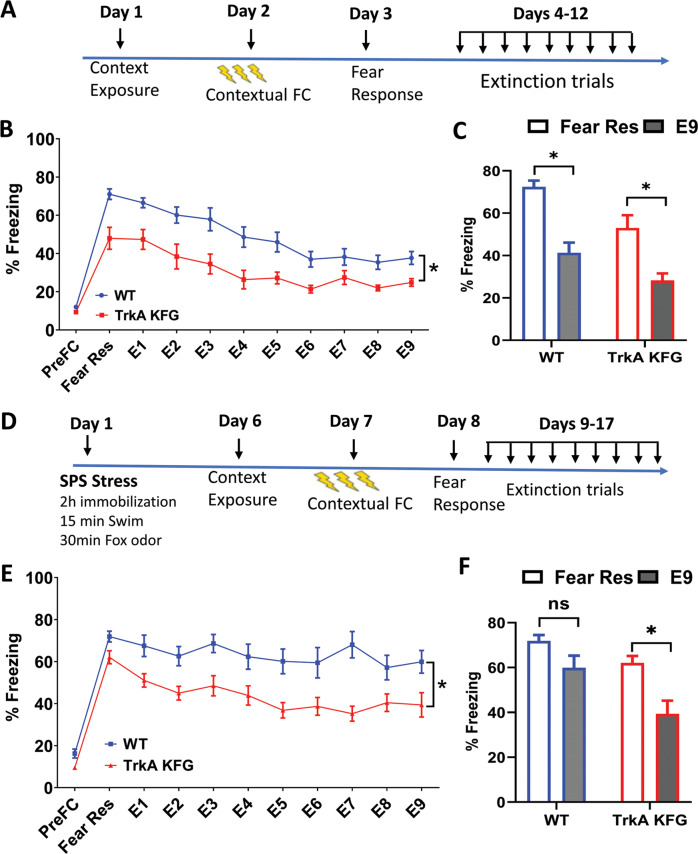
TrkA-KFG mice show attenuated freezing behavior in contextual fear conditioning and extinction. **A** Schematic representation of the protocol used to test fear response and extinction after contextual conditioning. **B** Quantification of the freezing response after contextual conditioning shows a reduced expression of fear (Fear Res as in day 3 in **A**) by TrkA-KFG mice (% Freezing 72.43 ± 2.95 in WT and 53.04 ± 5.98 in TrkA-KFG) that is maintained over the course of 9 extinction trials (E1–E9 day 4–12 in **A**) compared to WT mice. *N* = 13 for WT and 12 for TrkA-KFG mutants. **C** Graphical representation of the difference in freezing response measured as difference between the original fear response and last extinction trial (Points Fear Res vs E9 in panel **B**). Note that both WT and TrkA KFG mice show significant, but similar reduction in fear response at the end of the extinction trials (% Freezing 72.43 ± 2.95 vs 41.33 ± 4.79 in WT mice and 53.04 ± 5.98 vs 28.33 ± 3.23 in TrkA-KFG mice. Data are the Mean ± SEM, *p* < 0.05 Mann–Whitney Test). **D** Schematic representation of the protocol used to test the fear response and extinction in the SPS stress model. **E** In the SPS model of PTSD-like behavior, 6 days after stress, the mice were subjected to fear conditioning and extinction protocol as in **C**. Similarly to the results obtained with non-stressed animals in **B**, TrkA-KFG mice show attenuated fear response and reduced freezing during the extinction trials when compared to control mice. % Freezing at each timepoint was analyzed by Mann–Whitney Test. *p < 0.05; *n* = 10 for WT and *n* = 11 for TrkA-KFG mice. **F** Graphical representation of the difference in freezing response between the original fear response and the last extinction trial (Points Fear Res vs E9 in panel **E**) in WT (% freezing 71.92 ± 2.59 vs 59.99 ± 5.43, *p* > 0.05 n.s.) and TrkA KFG mice (% freezing response 62.08 ± 3.09 vs 39.39 ± 5.82, Data are the Mean ± SEM, *p* < 0.05 Mann–Whitney Test). Note that only the TrkA KFG mice show a significant reduction in fear response during the extinction trials.

NGF-TrkA signaling plays a critical role in the establishment of functional BFCN innervation to the hippocampus and the septo-hippocampal circuit is widely recognized as an important regulator of information processing by the hippocampus. Moreover, since the hippocampus and cholinergic signaling are important for contextual fear encoding and extinction [13–15], processes that are impaired in PTSD patients [40], we tested mutant and control mice in contextual fear conditioning and extinction paradigms (Fig. 3). On day 1, when mice were introduced to the context in a 3-min session, baseline freezing behavior was similar between the groups (pre-fear conditioning, PreFC point in Fig. 3B). When measured for their fear response 24 h post conditioning, TrkA-KFG mice showed reduced fear response, as measured by reduced freezing time when compared to WT mice (Fear Res point in Fig. 3B). This is similar to what we observed in the initial behavioral screen (Fig. 2B), further reinforcing the notion that increased TrkA-cholinergic signaling in TrkA-KFG mutant mice leads to an attenuation of fear encoding. After measuring the fear response, we subjected the mice to 9 fear extinction trials (one per day) by exposing the mice to the same context for 3 min but without the foot shock (E1-E9, Fig. 3B). During those 3 min, TrkA-KFG mice maintained the significantly lower freezing behavior in all the extinction trials when compared to WT mice (Fig. 3B, C).

While the fear conditioning paradigm measures normal fear response, modeling PTSD-like behavior requires exposure to a stressful or traumatic event that results in maladaptive (exaggerated fear and/or deficient extinction) response [27, 29, 41, 42]. Therefore, we expanded our analysis by employing a modified single prolonged stress (SPS) model to induce PTSD-like behavior in mice (Fig. 3D schematic). Single prolonged stress was caused by subjecting the mice to 2 h immobilization followed by 15 min of forced swim stress and 30 min exposure to TMT-fox odor. Seven days later, mice were evaluated for contextual fear conditioning and extinction. Importantly, while the control mice did not show a significant reduction in freezing from the initial fear response value during the extinction trials, the TrkA-KFG mice subjected to SPS showed significant reduction in fear response during the extinction phase (Fig. 3E, F). These data strongly suggest that the beneficial effects on fear processing in TrkA-KFG mice is enhanced in situations causing PTSD-like behavior.

### The GABA antagonist picrotoxin rescues the reduction in fear response and extinction of TrkA-KFG mice

**Fig. 4:**
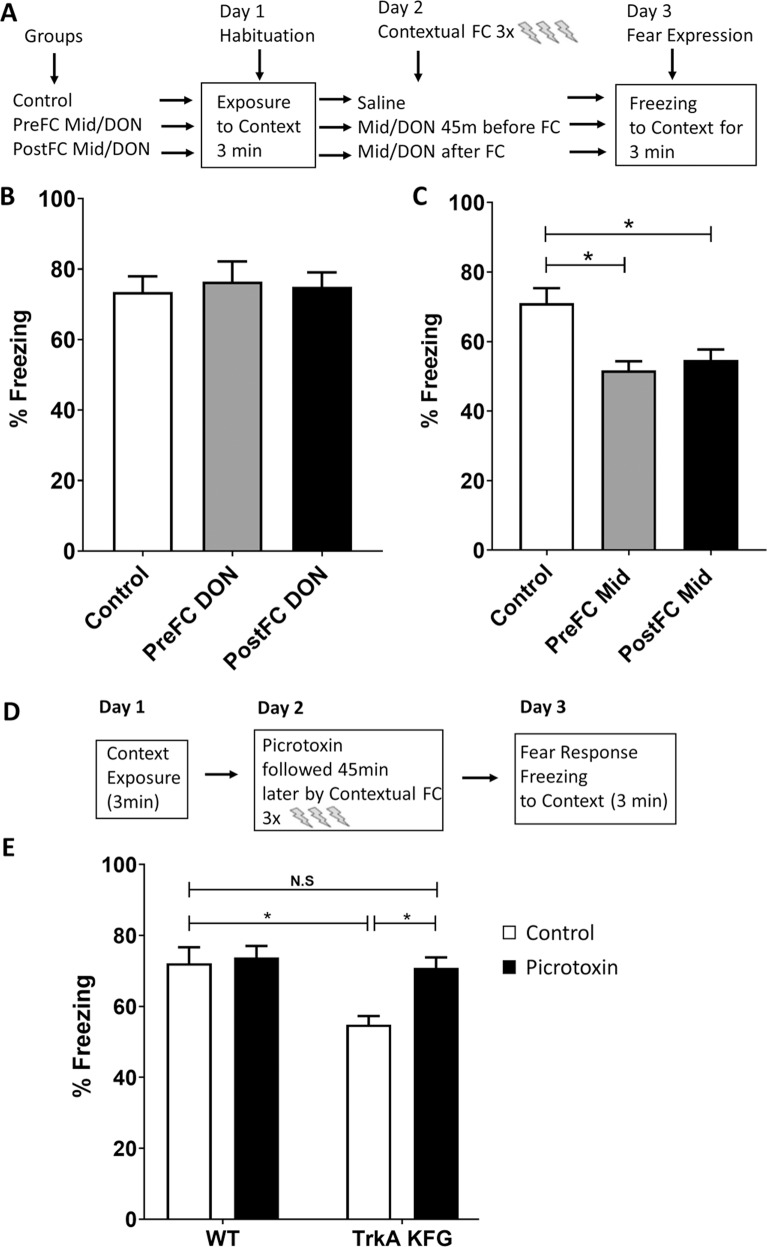
GABAergic signaling regulates acute contextual fear response. **A**, **D** Schematic representation of the protocols used to test GABAergic and cholinergic signaling role in contextual fear response. **B**, **C** Quantification of the freezing response of WT mice treated with donepezil (DON, **B**) or midazolam (Mid, **C**) 45 min before (PreFC DON or PreFC Mid) or immediately after (PostFC DON or PostFC Mid) contextual fear conditioning showing that augmentation of GABAergic signaling with midazolam reduces fear response when injected either before or immediately after contextual fear conditioning. *p < 0.05, non-parametric ANOVA followed by Dunn’s multiple comparison test, *n* = 8/group). **E** The GABA antagonist picrotoxin injected (0.5 mg/kg intraperitoneal) 45 min before fear conditioning restores the fear response of TrkA-KFG mice to WT control levels. *N* = 8–10/group. Note that picrotoxin, per se, does not alter the fear response in WT animals. *p < 0.05, Two-way ANOVA followed by Tukey Multiple comparison test.

We have previously reported that an increase in TrkA signaling in DRG neurons leads to hypersensitivity to nociceptive stimuli in TrkA-KFG mice [20], which makes our current finding of reduced fear response unexpected and surprising (Fig. 3). In fact, if TrkA-KFG mice are hypersensitive to pain they should display increased fear response to a painful stimulus. One major difference between DRG nociceptive neurons and basal forebrain cholinergic neurons is that while DRG neurons are excitatory neurons releasing mainly glutamate, BFCN co-release both excitatory (acetylcholine) and inhibitory (GABA) neurotransmitters [43, 44], and GABAergic signaling plays a role as important as acetylcholine in the encoding of fear and control over intrusive thoughts. Moreover, hippocampal cholinergic projections also establish GABAergic synapses suggesting that increased TrkA expression and cholinergic activation in TrkA KFG mutant mice may influence post-synaptic GABAergic neurons which in turn would result in heightened activity of inhibitory neurons [45–47]. To dissect a possible role of cholinergic versus GABAergic signaling in mediating altered fear response we employed a pharmacological approach. To this end, we treated WT mice with the cholinesterase inhibitor donepezil to augment cholinergic signaling or the GABA agonist midazolam to enhance GABAergic signaling (Fig. 4A). On day 1, WT mice were introduced to the context in a 3-min habituation session (“Control” in Fig. 4B, C). On day 2, mice were injected with donepezil (3 mg/kg i.p) or midazolam (1.25 mg/kg i.p) either 45 min before (PreFC) or immediately after (PostFC) contextual fear conditioning. On day 3, animals were tested for fear response by scoring freezing time over 3 min (Fig. 4B, C, PreFC and PostFC). Interestingly, administration of donepezil did not show any effect on encoding of fear (Fig. 4B) whereas midazolam significantly attenuated the fear response (Fig. 4C) when administered either before (27% decrease) or immediately after (23% decrease) the fear conditioning. These data suggest a significant GABAergic signaling role in the regulation of acute fear response after fear conditioning in agreement with earlier reports [23, 48]. To further test whether a change in GABA signaling may be part of the mechanism causing the attenuation in fear response in the TrkA-KFG mutants we used a low dose (0.5 mg/kg i.p) of the GABA antagonist picrotoxin 45 min before fear conditioning (Fig. 4D). As expected, the low dose picrotoxin did not change the baseline fear response in WT animals. However, pretreatment of the TrkA-KFG mice with picrotoxin restored the fear response to WT levels (Fig. 4E).

### Donepezil enhances fear extinction in a mouse model of PTSD-like behavior

**Fig. 5:**
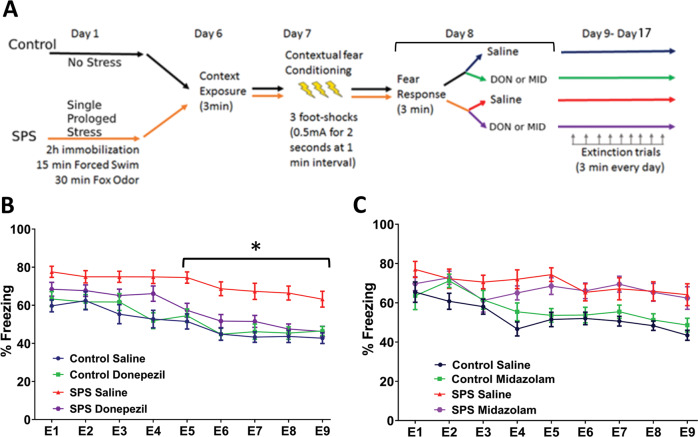
Donepezil augments fear extinction in a mouse model of PTSD-like behavior. **A** Schematics diagram of the SPS model used for contextual conditioning and extinction paradigm. Freezing time to context after induction of fear in WT mice was measured for 9 consecutive days in extinction-learning trials whereby the animals were re-exposed to the context for 3 min/day without receiving the foot shock. **B** Donepezil treatment during the extinction trials reduces freezing behavior in the SPS model. WT mice were treated each day with donepezil starting at day 8 after measuring the fear response and each following day immediately after each extinction trial. Note the overall higher freezing time in saline-treated stressed group. In contrast, donepezil-treated SPS group mice could extinguish their fear from the 5th extinction trial onwards similarly to the non-stressed animals. *p < 0.05, *n* = 18–22/group, statistical analysis by two-way ANOVA followed by Tukey’s post-hoc multiple comparison test. **C** Treatment of animals with the GABA agonist midazolam does not affect fear extinction relative to the SPS saline group (*n* = 8–12/group). Note that neither donepezil nor midazolam affects extinction learning in condition of non-stress (Control Donepezil in **B** and Control Midazolam in **C** relative to Control Saline).

While our results so far imply a significant role of NGF-TrkA signaling in regulating PTSD-like behavior, there are a few issues that we still need to consider. For example, the TrkA-KFG mutation is present throughout ontogenesis and therefore we cannot exclude that the behavioral changes are caused by developmental rather than functional neuronal changes. The lack of obvious anatomical changes in the BFC system of adult TrkA-KFG mice and the ability to rescue the TrkA-KFG phenotype with doses of picrotoxin that do not affect the normal behavior of WT mice so far suggest that the changes may be functional. Most importantly, what is the translational significance of this finding? To gain insight into these questions, we employed a pharmacological approach by using donepezil as a means to enhance cholinergic signaling or midazolam to promote GABAergic signaling in a SPS model of PTSD-like behavior in WT mice. Importantly, we started the drug treatments after the fear-expression phase to identify their role specifically on extinction learning. A group of WT animals was used as no stress saline controls while another group was subjected to single prolonged stress including 2 h immobilization, followed by 15 min forced swim and 30 min exposure to predator odor (Fig. 5A, schematic). On days 6 and 7, the animals were subjected to context exposure and fear conditioning, respectively. Fear response was measured on day 8. After fear response evaluation, a subgroup of animals in the stressed as well as non-stressed group was treated with either donepezil (3 mg/kg/day i.p) or midazolam (1.25 mg/kg/day i.p). From day 9 to 17, all groups received daily extinction trial. Drug or saline injections were performed once every day immediately after the trial throughout the course of the extinction trials period. As expected, non-stressed animals (saline as well as donepezil treated) exhibited a gradual decrease in freezing behavior (Fig. 5B). In contrast, saline-treated SPS group mice exhibited a significant deficit in fear extinction over the course of all extinction trials when compared with non-stressed mice (Fig. 5B). Interestingly, while the freezing behavior in donepezil treated SPS mice was similar to that of SPS saline group during the first 4 extinction trials, from the 5^th^ trial onwards, there was a gradual and significant decrease in freezing similar to that of non-stressed animals. To test whether donepezil, per se, may have an effect on locomotor activity or anxiolytic effects that may mask its effect on learning we performed an open field analysis of WT mice after acute (single dose) and chronic (9 daily injections) donepezil or saline injections. On all parameters of mobility, donepezil treated mice performed similarly to control, saline-injected mice suggesting that donepezil does not have anxiolytic effect or affect motor activity but enhances fear extinction through learning (Supplementary Fig. 7). In contrast, midazolam did not affect extinction learning in SPS mice when compared to saline-treated SPS mice (Fig. 5C). These data indicate that experience of stress induced by SPS results in PTSD-like behavior as reflected by deficient extinction learning. However, following SPS, continued treatment with a cholinergic agonist to selectively increase cholinergic signaling enhances extinction learning.

## Discussion

In this study, we report that enhanced TrkA-mediated cholinergic neurotransmission reduces fear response and enhances fear extinction. While testing the hypothesis that augmenting TrkA-cholinergic signaling could be of relevance to treating PTSD-like behavior, we also found that pharmacological augmentation of cholinergic signaling using donepezil, a cognition enhancing drug that promotes brain cholinergic signaling, accelerated fear extinction in an animal model of PTSD-like behavior.

Both animal and human studies have shown that acute as well as chronic physical or psychological stress alters plasma NGF levels [49, 50]. While it is not clear if these changes contribute to the behavioral outcome after stress or to the pathophysiology of PTSD, it is well established that NGF-TrkA signaling has a role in neuroplasticity and cognitive flexibility [16, 17] and plays a critical role in establishing the septo-hippocampal circuitries that affect hippocampus-dependent contextual fear encoding [13–15]. The dissection of these circuitries have been aided by the use of fear conditioning paradigms in rodents which have been useful not only to evaluate fear responses but also to model extinction-based exposure therapy used in clinical psychotherapy for PTSD patients [51].

In the CNS, the TrkA-KFG mouse model exhibits elevated levels of TrkA protein and acetyl choline synthesizing enzyme ChAT and therefore provides a gain-of-function model of NGF-TrkA signaling. This gain of function is also present in the PNS and leads to hypersensitivity to painful stimuli [20]. Therefore, a reduction in fear expression following the painful stimulus used in the fear response paradigm was somewhat surprising. The basal forebrain cholinergic region is a neurochemically diverse population of neurons expressing GABA, acetylcholine, or even co-expressing and co-releasing both neurotransmitters [44, 52]. Manipulations of this region have led to counterintuitive observations probably because of this neurotransmitter heterogeneity. For example, optogenetic stimulation of cholinergic fibers results in GABA-mediated inhibitory response in postsynaptic cells [43]. Pharmacologically, the α7-nAChR agonist FMR17848 and the cholinesterase inhibitor donepezil both increase frequency and amplitude of inhibitory GABA currents in septo-hippocampal slices in addition to increasing LTP [53]. Moreover, hippocampal cholinergic projections have been shown to establish GABAergic synapses and, septo-hippocampal activation yields composite results secondary to increased firing of inhibitory interneurons and decreased firing of principle cells that are relevant for encoding of fear [45, 52, 54, 55]. In addition to these data, behavioral studies have also been in line with our results. Hippocampal theta rhythm plays an important role in information processing such as fear encoding and retrieval, and is regulated by septo-hippocampal projection [56]. Interestingly, theta stimulation of hippocampus that mimics septo-hippocampal rhythm, unexpectedly, reduced the contextual fear response [57]. Interestingly, it has been reported that exogenous administration of NGF attenuates PTSD-like symptoms in a SPS model of PTSD-like behavior [58]. Taken together, these observations suggest that in conditions of stress/challenge causing septo-hippocampal activation, such as the one induced by fear conditioning paradigms, basal forebrain neurons may co-release GABA and acetylcholine. Since GABA is a fast inhibitory neurotransmitter, it probably plays a predominant role in encoding and/or expression of fear, which is why the attenuation of fear in TrkA-KFG is mimicked by the GABA agonist midazolam and blocked by the GABA antagonist picrotoxin. In contrast to GABA, acetyl choline is a slow acting neuromodulator [59], which could explain why the increase in ChAT levels seen in the mutant mice and mimicked by treating WT mice with the acetyl cholinesterase inhibitor donepezil, plays a more pronounced role during the fear extinction learning in the SPS model of PTSD-like paradigm. Alternatively, the interaction of cholinergic and GABA systems could be entirely postsynaptic to the BFCN terminals. NGF is known to be expressed in a subset of inhibitory neurons of the hippocampus **[**47] and hippocampal cholinergic projections have been shown to establish GABAergic synapses. In this scenario, in the TrkA-KFG mice, the increased TrkA-mediated cholinergic activation would lead to enhanced NGF release from the interneurons, leading to a positive, trophic effect on the BFCN while simultaneously increasing the activation of the inhibitory GABAergic neurons suppressing the activity of pyramidal neurons. While we cannot presently pinpoint the precise reasons affecting the flow of information through the cortical, hippocampal and/or amygdala circuits that regulate association foot-shock to context and/or tone, it is somewhat surprising that we could pharmacologically rescue and reproduce this complex phenotype respectively in TrkA-KFG and wild-type mice.

However, while the pharmacological approach provides strong translational relevance to the findings, systemic administration of the drug does not allow for the unambiguous identification of the target and circuitries. Future studies employing local drug application in specific brain regions, surgical ablation, and selective chemo- and/or opto-genetic manipulations of the cholinergic circuits will help dissect the mechanisms underpinning the behavioral changes. Nevertheless, our genetic model has led us to the identification of a possible new avenue for the treatment of PTSD. Because of the translational focus of our study, we have limited our analysis to identifying a possible role of cholinergic signaling in promoting extinction learning. For this we have used donepezil post-fear expression to identify a corrective, rather than preventive, strategy in promoting extinction learning in animal models of PTSD-like behavior. To date, exposure-based therapy (CBP) represents the gold standard in PTSD treatment and it relies on the exposure of the patient to stimuli that generate fear with the goal to overcome or lower the magnitude of the response [60]. Fear extinction paradigms in animals employ a similar approach whereby the animals learn to confront the fear-inducing stimuli and gradually decrease their fear levels. Therefore, the extinction trials used in the present study mimic the exposure-based learning in clinical settings. Since the extinction-promoting effect of donepezil increased during the course of the extinction trials and PTSD is a chronic disease, these observations identify the therapeutics potential of using donepezil in conjunction with cognitive behavioral therapy. While the impact of cognition enhancers such as the use of the NMDA agonist d-cycloserine or HDAC inhibitors has been recognized and is investigated as an adjuvant to exposure-based therapies [7, 9, 61, 62] it is surprising that donepezil has not yet been used for managing fear extinction, despite being available for more than two decades for its cognition promoting effects in patients with dementia. However, a clinical study based on off-label use of donepezil describes four cases where donepezil treatment relieved nightmare associated with PTSD [63]. Thus, despite being limited, these data are encouraging. Intriguingly, there are also two case reports where the use of donepezil for treatment of dementia has resulted in the recall of traumatic memories [64, 65]. One of the serious challenges for exposure therapies of PTSD patients is that maladaptive memories are difficult to overcome as they have been stabilized or consolidated over a period after the original traumatic experience. The return of fear, therefore, occurs frequently even after the successful initial exposure therapy. Nevertheless, recent literature has demonstrated that memory reactivation may, in fact, cause the previously fixed traumatic memories to enter a labile phase rendering them more amenable to manipulations aimed at promoting safety-learning during reconsolidation [66]. Therefore, these two case reports raise the possibility that donepezil can additionally target the process of memory reactivation which, in turn, would allow better targeting of the reconsolidation phase of fear memories during the course of exposure therapy.

In summary, our data provides evidence that TrkA-cholinergic signaling regulates fear response and the pharmacological targeting of this system has the potential to be used in conjunction with psychotherapy to correct the inability to extinguish fear. Since donepezil is a drug enhancing cholinergic signaling, it is orally bioavailable, safe and FDA approved for the treatment of Alzheimer’s dementia [67] it would be important to consider this cognitive enhancer for extinction learning during exposure therapy in PTSD patients.

## Supplementary information

Supplememntary Material

Supplementary Figure 1

Supplementary Figure 2

Supplementary Figure 3

Supplementary Figure 4

Supplementary Figure 5

Supplementary Figure 6

Supplementary Figure 7
