# Supplementary material for: TrkA-cholinergic signaling modulates fear encoding and extinction learning in PTSD-like behavior
Source: Transl Psychiatry. 2022 Mar 17;12:111. doi: 10.1038/s41398-022-01869-2 (PMC8931170; doi:10.1038/s41398-022-01869-2)
Supplement: Supplementary file 1 — Supplememntary Material [file 41398_2022_1869_MOESM1_ESM.docx]

**Supplementary Information**

**TrkA-cholinergic signaling modulates fear encoding and extinction learning in PTSD like behavior.**

Sudhirkumar Yanpallewar, Francesco Tomassoni-Ardori, Mary Ellen Palko, Zhenyi Hong, Erkan Kiris, Jodi Becker, Gianluca Fulgenzi and Lino Tessarollo

**Supplementary Materials and Methods:**

**Immunohistochemistry and stereology:**

Animals were perfused with 1X PBS followed by 4% paraformaldehyde (4% PFA). Brains were isolated, post-fixed in 4% PFA overnight and cryoprotected in 30% sucrose before sectioning. Serial 50 um brain sections were cut on a cryostat. One every third section (6 in total) per animal containing the BFCN region (medial septum, vertical and horizontal diagonal band of Broca; 1.10  to 0.14 mm anterior to Bregma- Paxinos and Franklin’s Mouse Brain Atlas, 2001) was used for ChAT immunohistochemistry. The merging of the two sides of the corpus callosum at 1.10mm and anterior commissure at 0.14 mm anterior to Bregma were used as anatomical landmark to collect the sections. Briefly, after a blocking treatment in 10% horse serum in 1X PBS with 0.3% Triton X100 for 2h, the sections were incubated first with a goat anti-ChAT antibody (1:250, Millipore) overnight at RT and then with a Horse biotinylated anti-goat antibody (1:500, Invitrogen). The signal was amplified with an avidin-biotin signal amplification reagent and detected with DAB substrate. The total number of ChAT+ cholinergic neurons in BFCN was calculated for each animal. For calculation of the cell area and cell volume, 80 - 120 ChAT+ cholinergic neurons per animal were subjected to a nucleator method analysis using stereoinvestigator software (MBF Bioscience, USA).

**TrkA mRNA in situ hybridization**

A TrkA-specific riboprobe that spans 445 nucleotides of the extracellular domain of the mouse TrkA (amino acids 259-460) was cloned into the pGEM7Zf+ plasmid and used to generate sense and anti-sense DIG (digoxigenin)-labelled riboprobes. RNA probes were labelled with DIG-UTP by in vitro transcription with SP6 or T7 RNA polymerase using the Riboprobe in vitro transcription systems (Promega, USA) following the manufacturer’s protocol. Free floating coronal brain sections (50 μm thick) in 12 well plates were proteinase K treated (5 μg/ml) in PBS and postfixed in 4% PFA. Sections were then incubated in hybridization buffer (50% formamide, 5× SSC, 50 μg/ml yeast tRNA, 50 μg/ml heparin, and 0.1% Tween 20) for 1 h at 70°C, before an overnight incubation with the sense or anti-sense probe at 70°C in the same buffer. After several post-hybridization washes (2x30 min in 50% formamide, 5SSC at 70°C; 2x 30 min in 0.1 M Tris-HCl, 0.5 M NaCl, 0.1% Tween 20 at RT; 2x 30 min in 50% formamide, 2XSSC at 70°C) and incubation for 2 h at RT in blocking solution (10% normal goat serum, 2 mM levamisole in 0.1M Tris-HCL pH 7.5), sections were incubated overnight at 4°C with alkaline phosphatase-conjugated anti-Dig antibodies (1:1000; Roche, USA). Following several washes with 1X TBST, sections were incubated twice for 10 min in NTMT buffer and then in NBT/BCIP substrate (Roche) at RT to develop the hybridization signal. After an overnight air drying, slides were then cover slipped with Cytoseal XYL mounting media (Thermo Scientific, USA).

**Behavioral analysis:**

**Open field test:** The general locomotor activity was evaluated in an open field chamber (16 x 16-inch Plexiglas Chamber, Omnitech Electronics USA). Animals explored the arena for 15 min and infrared sensors mounted on to the walls of the chamber recorded the total distance traveled, immobility time, rearing, ambulation, and time spent in the center part of the field.

**Rota rod test:** To determine the muscle strength and coordination we used an accelerating rota rod apparatus (Ugo Basile) as previously described (Yanpallewar et al., 2012). Animals were placed on a rotating rod that accelerated from 5 to 40 rpm over a 5 min period. For each mouse, three individual trials were conducted separated by 1 h and the average of the time spent on the rotating rod from the three trials was used for comparison between genotypes.

**Elevated Plus Maze:** Anxiety related behavior was assessed using a Biobserve Elevated Plus Maze Apparatus (Germany). Briefly, animals were placed in the center of a plus maze with two open arms (10 x 40 x 50cm) and two closed arms (50 x 40 x 8 cm) at a height of 50 cm. A video tracking system recorded the number of crosses between the arms and the time spent in the open arm for each animal over a 10 min period.

**Forced Swim test:** Animals were tested in the Porsolt swim test in a 5L glass cylinder (diameter 185mm) for evaluation of depression-like behavior. Briefly, the animals were placed in the glass cylinder with water level up to 3L and, swimming, wall-climbing and floating (immobility) behavior was recorded with a video camera for 6 min. An observer blind to the genotype recorded the latency to immobility and total immobility time during the last 4 min for each animal.

**Startle response and pre-pulse inhibition (PPI):** Startle response and its PPI (the attenuation of a startle response when a non-startling stimulus/pre-pulse is presented before a startling stimulus) was used as a functional measure of sensorimotor gating. We used a MED-ASR-PRO1 startle testing system (MedAssociates Inc, USA) to evaluate the startle response and PPI. The apparatus consisted of a 50.8 cm x 33.0 cm x 30.5 cm sound-attenuating chamber. During the testing, the mice were placed in a Plexiglas cylinder (3.2cm internal diameter) resting on top of a sensitive device that records vibrations that occur as result of the startle response. The acoustic startle stimulus and the pre-pulse stimulus were delivered via a high-frequency speaker, placed within the sound-attenuating chamber. After placing in the cylinder, the individual mice were allowed a 5-min adaptation period before the start of the session. The session was initiated with ten 120 dB, 38 msec sound bursts. Exposure to these bursts allowed for the establishment of a startle response and the average startle response between the genotypes was compared. Following the measurement of the startle response the animals were subjected to a 28-trial session where the stimuli were presented in a pseudo-random order. These trials consist of four null trials (no exposure to sound), four 120 dB (each of 38ms) trials to measure the acoustic startle response and 16 trials comprising of a pre-pulse of either 74, 78, 82 or 86db intensities presented for 18 ms prior to a startle pulse of 120 dB to measure the inhibition of startle response. Additionally, one each pre-pulse only trial was conducted at 74-, 78-, 82- and 86-dB levels. Throughout the experiment, the inter-trial interval varied randomly between 10–20s. Percentage PPI (%PPI) was calculated at each pre-pulse stimuli and compared between genotypes.

**Auditory Cued Fear Conditioning:** Fear conditioning to the auditory cue was performed in an automated system. The mice were placed in the fear conditioning chamber (Coulbourn Instruments, USA) and allowed exploration for 2 min followed by 85 dB tone [conditioned stimulus (CS)] for 30 seconds. The tone co-terminated with a mild footshock [unconditioned stimulus (US); 0.5 mA current, 2 Second duration] delivered through the gridded electric floor. Animals were then subjected to two more CS–US pairings with 30 s intertrial interval; they were then returned to home cage after a further 30 sec in the conditioning chamber. 24 h after training the mice were evaluated for cued conditioning. For the cued response measurement, the context was changed by conducting the test in a different chamber (Omnitech electronics Open field apparatus), replacing the electric grid with a solid plexiglass floor, different wall pattern and light intensity and using a Lavender scent. Animals were allowed to explore this new context for 2 min, followed by 85 dB tone for a 2 min period. Freezing response to this CS (tone) was recorded as % freezing and compared between WT and TrkA KFG mice.

**Supplementary Figure Legends**

**Supplementary Figure 1.** Basal Forebrain cholinergic neurons develop normally in TrkA-KFG mice. A) TrkA mRNA expression in adult mouse brain; Dig-in situ hybridization staining for TrkA mRNA showing the characteristic expression of TrkA in the basal forebrain cholinergic regions. Note the well-defined and dense expression in MS, VDB and HDB cholinergic regions. In contrast, TrkA mRNA expressing nuclei are sparse and few in the NBM-SI region. TrkA mRNA is expressed abundantly in striatum whereas it is absent in cortex. MS-Medial Septum, VDB-Vertical diagonal band of Broca, HDB-horizontal diagonal band of Broca, NBM-nucleus basalis of Meynert (delineated in red), SI- substantia innominate, LV-lateral ventricles, ac-anterior commissure, cc-corpus callosum, IC-internal capsule, CPu-Caudate putamen, LGP-lateral globus pallidus, ML-molecular layer, GCL- granule cell layer, PFC-prefrontal cortex. B) Representative choline acetyltransferase immunohistochemical staining of BFCN in WT and TrkA-KFG mice. C) Histogram showing the stereological quantification of the number of cholinergic neurons in the BFCN of 3-month-old WT and TrkA-KFG mutant mice as stained with the anti-ChAT antibody in B. D, E) Histograms reporting the cell area (D; WT 213.3± 7.20 vs TrkA-KFG 221.6 ± 7.91) and cell volume (E; WT 2556± 143.6 vs TrkA-KFG 3005 ± 146.5) of ChAT-positive neurons. Note the lack of differences in all parameters analyzed between TrkA-KFG and WT animals. Data are expressed as Mean ± SEM, n=4-5 mice/genotype.

**Supplementary Figure 2.** Generation of the TrkB-KFG and TrkC-KFG mutant mice. (A) Schematic of the vector and strategy to target the TrkB locus. The replacement-type targeting vector consists of a 10.0 kb 129/SV mouse genomic fragment. Exon 11 was modified by removal of the 9 nucleotides encoding the KFG domain by PCR (asterisk). The pGKneobpA cassette flanked by loxP sites was placed in the intron downstream of exon 11. The pGK-thymidine kinase (TK) cassette was introduced as a negative selectable marker. Exons 9-11 are indicated. (B) Southern blot analysis of tail DNA from 3-week-old mice with a 3’ external probe showing the switch of the WT Hind III (H in panel A) fragment from 9.5 kb (WT) to a 4.1 kb restriction fragment (TrkB neo) after neo insertion due to the targeting of the TrkB locus. Removal of the neo gene causes a further decrease of the Hind III-generated restriction fragment to 2.5 Kb. The presence of the KFG deletion was verified by PCR analysis using one primer upstream and downstream of the KFG domain. (C) Representative western blot images showing ChAT and Actin, used as control, protein levels in basal forebrain cholinergic neuron (BFCN) area from control (WT) and mutant (TrkB KFG) mice. (D) Schematic representation of the strategy used to target the TrkC locus. A Single strand oligonucleotide of 100 bases containing Exon 11 modified by the removal of the 9 nt encoding the KFG domain and addition of a BglII restriction site was used to target fertilized embryos by the CRISPR/Cas9 technique. Exons 9-11 are indicated. (E) Southern blot analysis of tail DNA from 3-week-old mice with a 5’ external probe shows the switch of the WT Bgl II (B2 in panel E) fragment from 9.3 kb (WT) to a 4.3 kb restriction fragment (TrkC DKFG) after the targeting. The presence of the KFG deletion was verified by PCR analysis using one primer upstream and downstream of the KFG domain. (F) Representative western blot images showing ChAT and Actin, used as control, protein levels in the basal forebrain cholinergic neuron (BFCN) area. Note that ChAT levels in the basal forebrain are not changed in the mutant mice when compared with WT controls.

**Supplementary Figure 3. TrkA-KFG mice have normal locomotor activity, muscle strength and coordination.** (A-E) Quantification of control and TrkA-KFG mice exploratory behavior including total distance traveled (A), rearing (B), ambulations (C), immobility time (D) and time spent in center of the field (E) over a period of 15-min. Mean ± SEM; n=10 per group (F) Rota rod test; histogram showing the time spent by the mice on the accelerating rotating rod before falling (5-40 RPM over 5 min). Data are expressed as Mean ± SEM; n=15 for WT and n=13 for TrkA-KFG mice.

**Supplementary Figure 4. Evaluation of anxiety- and depression-related behavior in TrkA-KFG mice.** (A, B) Elevated plus maze; quantification of the time spent in the open arms (A) and total number of crosses between open and closed arms (B) observed over a 10-min period in the elevated plus maze. (C, D) Forced swim test; swimming behavior of WT and TrkA-KFG mice recorded over a period of 6 min and quantified as the latency to immobility (C, floating) and time spent floating versus swimming and climbing (D) in the last 4min of the trial. Data are expressed as Mean ± SEM, n=15 WT and 13 TrkA-KFG mice.

**Supplementary Figure 5. Sensorimotor function is preserved in TrkA-KFG mice.** Startle response (A) and pre-pulse inhibition (PPI) of the startle response (B) was evaluated as described in Methods. A) Animals were allowed to habituate to the sound-proof enclosure for 5min after which a 120dB sound was delivered and the magnitude of the startle was evaluated. B) Percent of the inhibition of startle response for WT and TrkA-KFG mice was evaluated at 4 different pre-pulse intensities (74, 78, 82 and 86 dB). Data are expressed as Mean ± SEM; n=15 WT and 13 TrkA-KFG mice.

**Supplementary Figure 6. TrkA-KFG mice have normal cued conditioning and ChAT levels in Amygdala and prefrontal cortex.** A) Western blot analysis showing ChAT protein levels in amygdala and prefrontal cortex (PFC). Actin was used as control for loading. B) Histogram showing the quantification analysis of the ChAT band intensity relative to Actin in both genotypes. N = 5 per genotype. (C) Histogram showing the result from the cued conditioning test. Mice were conditioned with 3 CS × US pairings and the auditory-cued fear memory was tested 24 after training. Data are presented as Mean ± SEM (n = 10). Note the lack of significant difference in freezing levels between the two genotypes (% Freezing 79.38 ± 3.40 in WT vs 83.62 ± 3.05 in TrkA-KFG).

**Supplementary Figure 7: Donepezil treatment does not affect mobility related tasks in WT mice.** WT animals were subjected to single dose (A: Acute Donepezil) or 9-day treatment (B: Chronic Donepezil) with the drug and their locomotor behavior in an open field chamber (Omnitech electronics, USA) was evaluated to determine possible effects of Donepezil on locomotor activity. Testing was conducted 24hr after donepezil injection. Total distance traveled, immobility time, ambulations, rearing and time spent in center of the field over a period of 15-min were quantified in Control and Donepezil-treated mice. Data are expressed as Mean ± SEM; n=7 each group.

**Supplementary Table 1: List of Antibodies Used in the Study**

| **Name** | **Catalog Number** | **Dilution** | **Vendor** |
| --- | --- | --- | --- |
| Rabbit anti-TrkA | AB-NO3 | 1:1000 | Advanced Targeting System |
| Goat anti-ChAT | AB144P | 1:500 | Millipore-Sigma |
| Anti-Digoxigenin AP | 11093274910 | 1:2000 | Roche |
| Donkey anti-Rabbit HRP | AP182P | 1:5000 | Millipore-Sigma |
| Donkey anti-Goat HRP | AP180P | 1:5000 | Millipore-Sigma |
| Mouse anti-actin HRP | SC-47778 | 1:5000 | Santacruz |
| Biotinylated Horse anti-Goat | BA-9500 | 1:500 | Vector Laboratories |
