# Supplementary figures and images for: TrkA-cholinergic signaling modulates fear encoding and extinction learning in PTSD-like behavior
Source: Transl Psychiatry. 2022 Mar 17;12:111. doi: 10.1038/s41398-022-01869-2 (PMC8931170; doi:10.1038/s41398-022-01869-2)

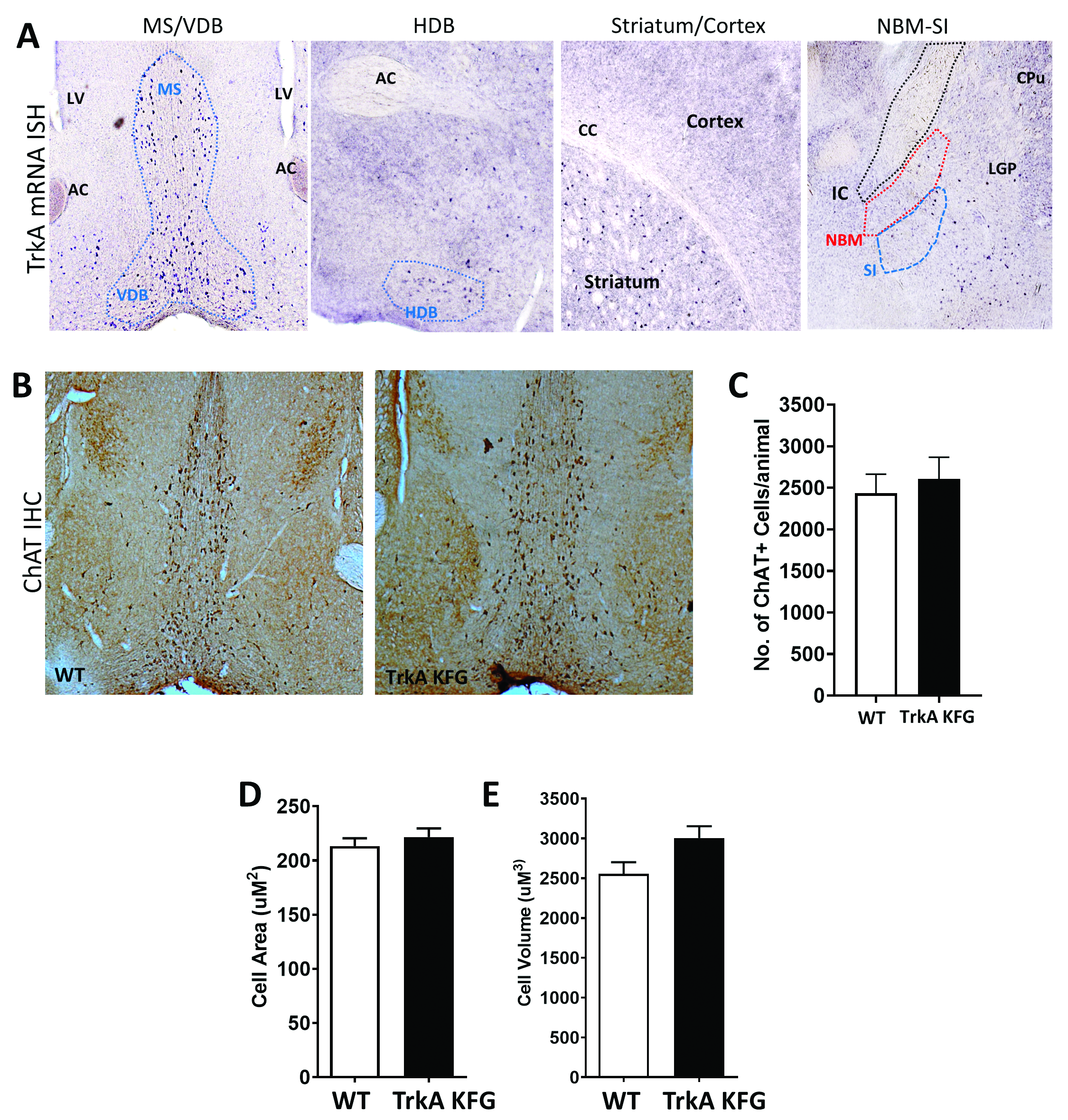

Supplement: Supplementary file 2 — Supplementary Figure 1 [file 41398_2022_1869_MOESM2_ESM.tif]

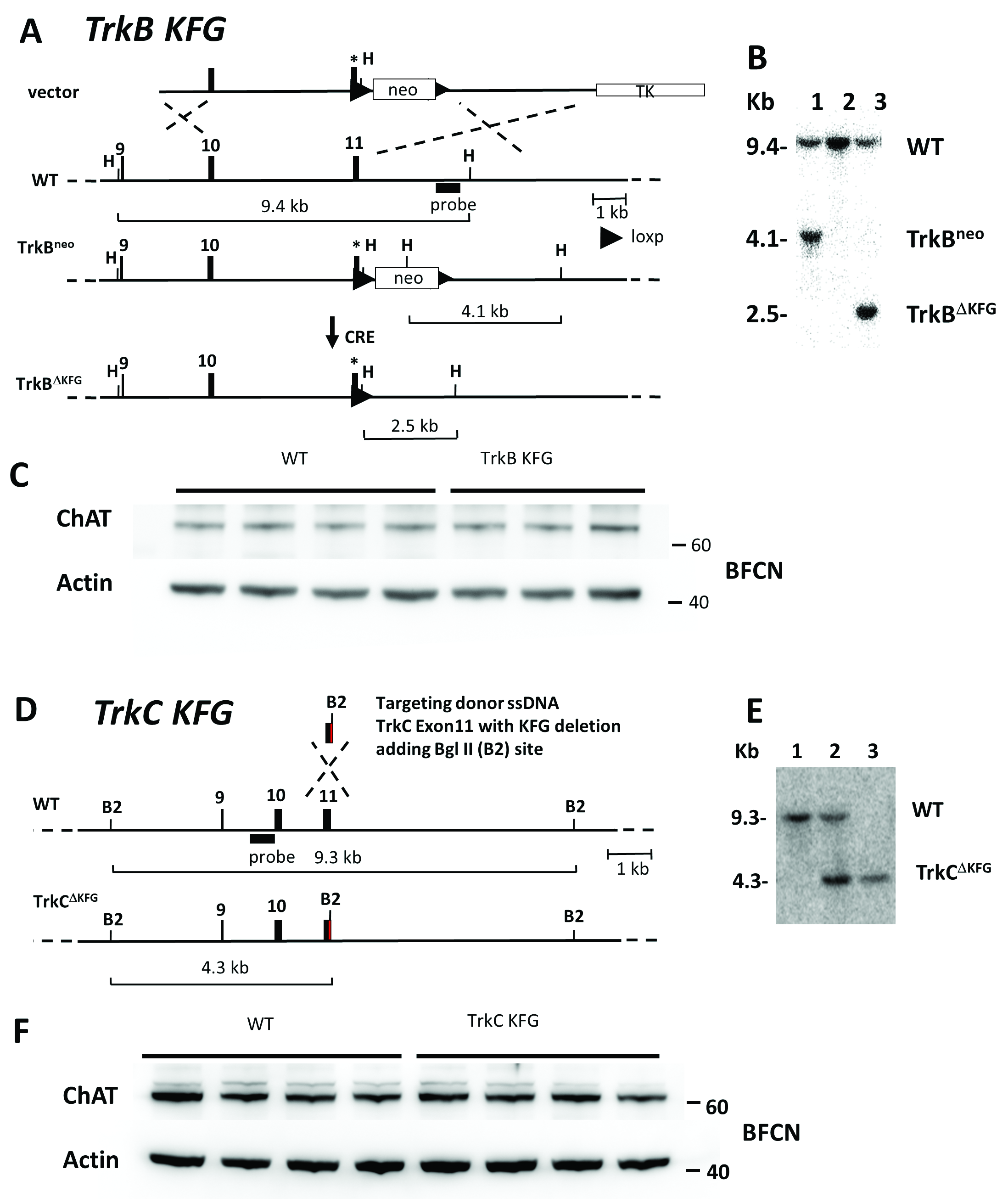

Supplement: Supplementary file 3 — Supplementary Figure 2 [file 41398_2022_1869_MOESM3_ESM.tif]

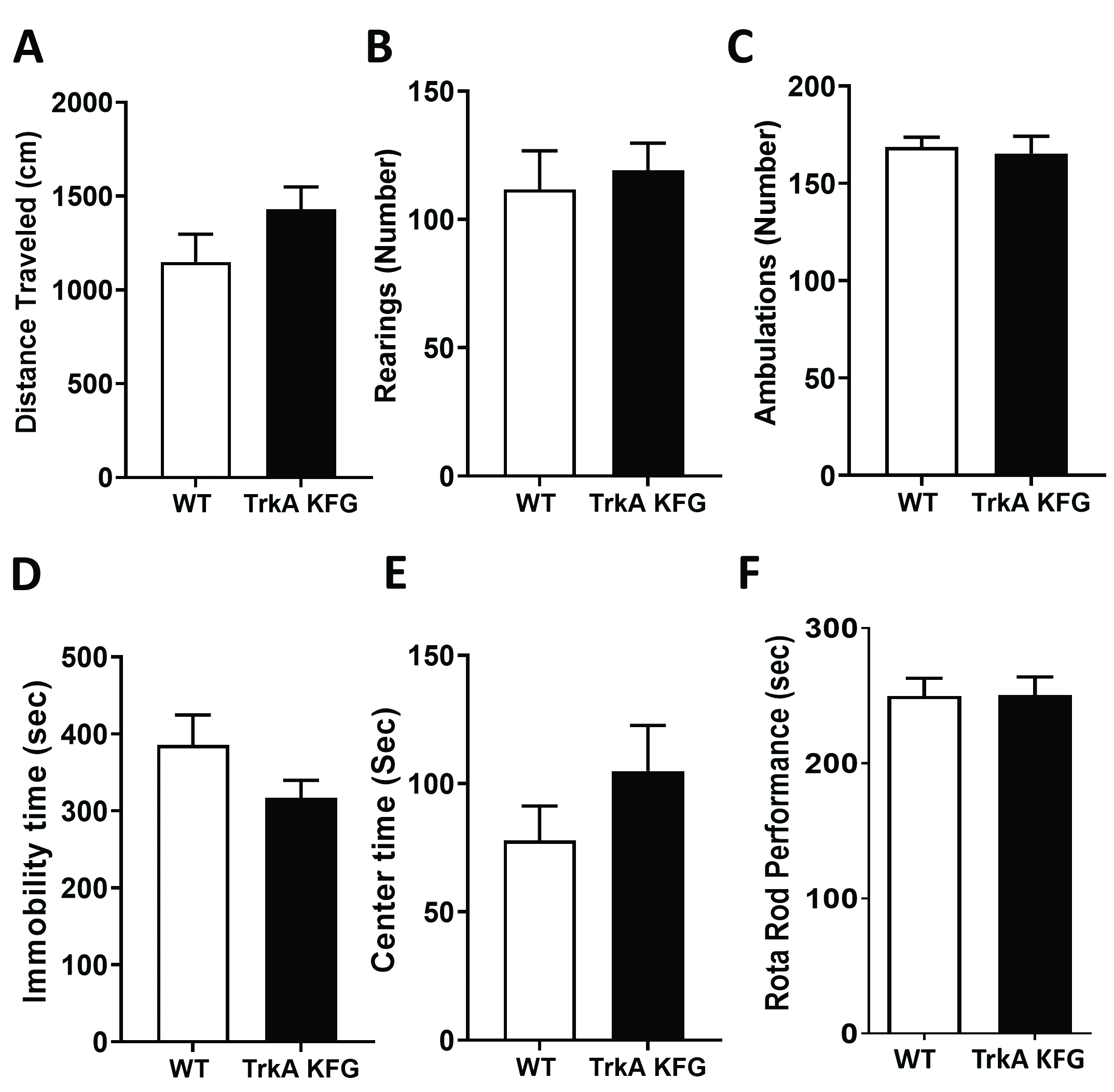

Supplement: Supplementary file 4 — Supplementary Figure 3 [file 41398_2022_1869_MOESM4_ESM.tif]

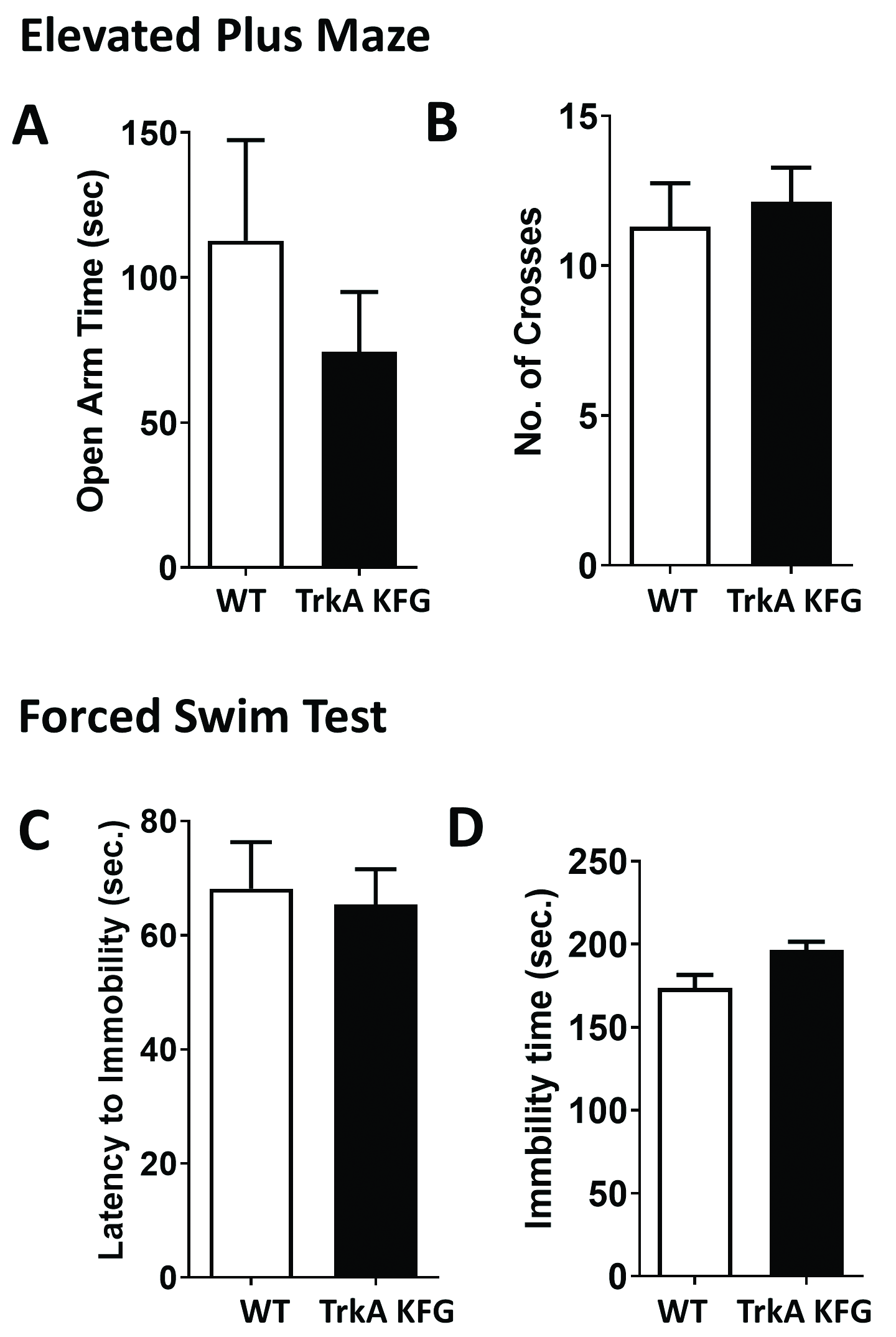

Supplement: Supplementary file 5 — Supplementary Figure 4 [file 41398_2022_1869_MOESM5_ESM.tif]

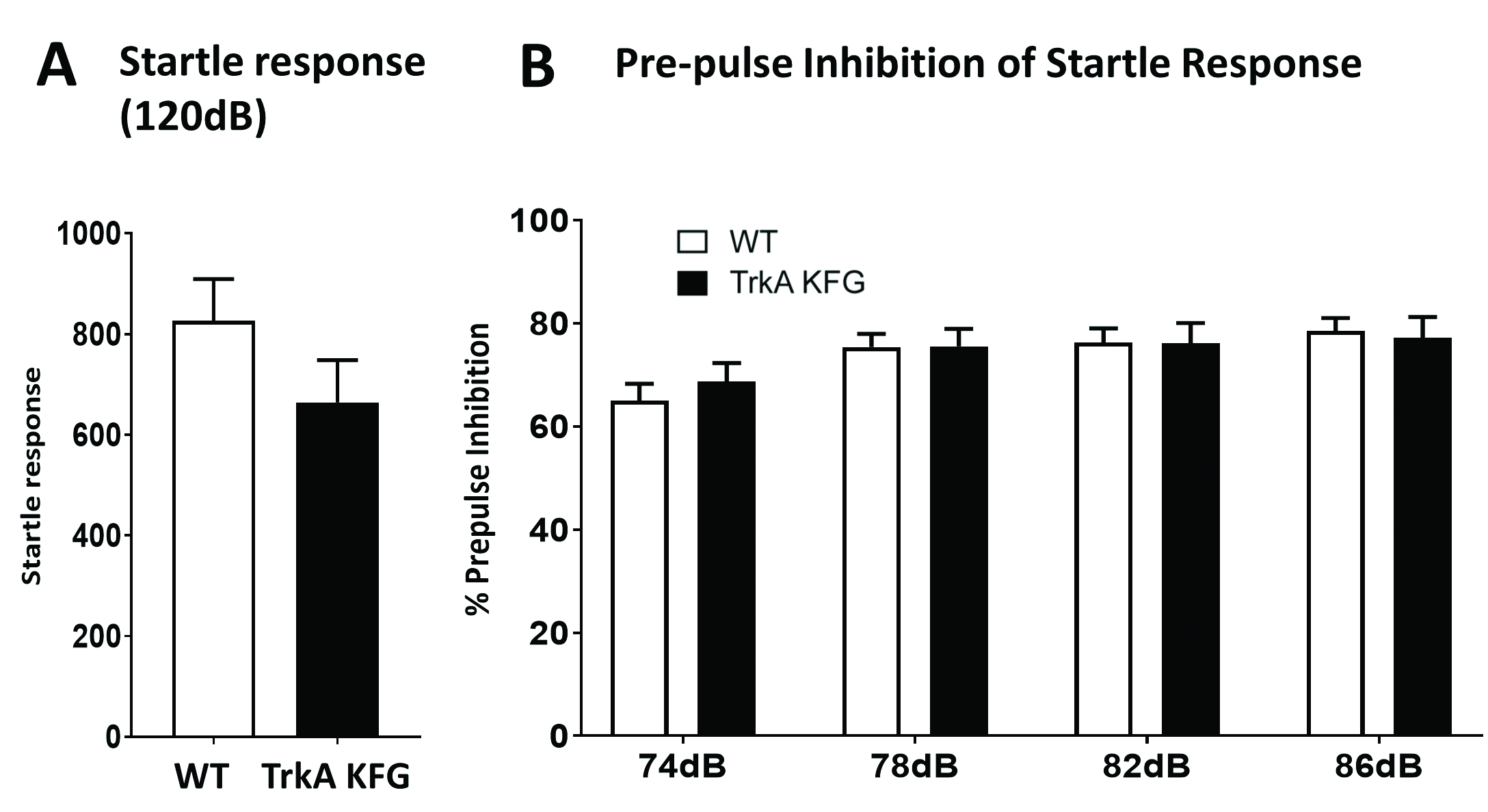

Supplement: Supplementary file 6 — Supplementary Figure 5 [file 41398_2022_1869_MOESM6_ESM.tif]

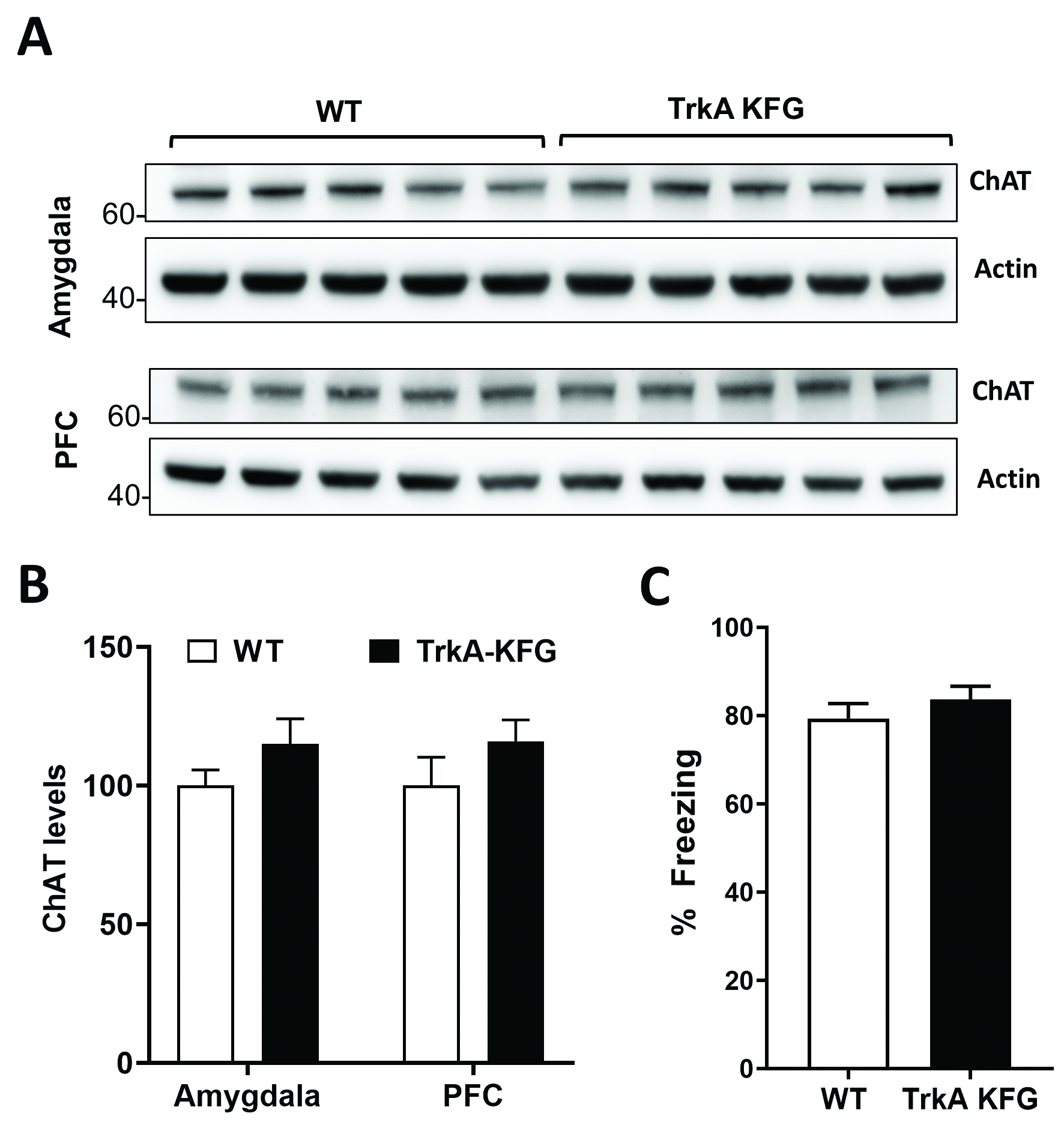

Supplement: Supplementary file 7 — Supplementary Figure 6 [file 41398_2022_1869_MOESM7_ESM.tif]

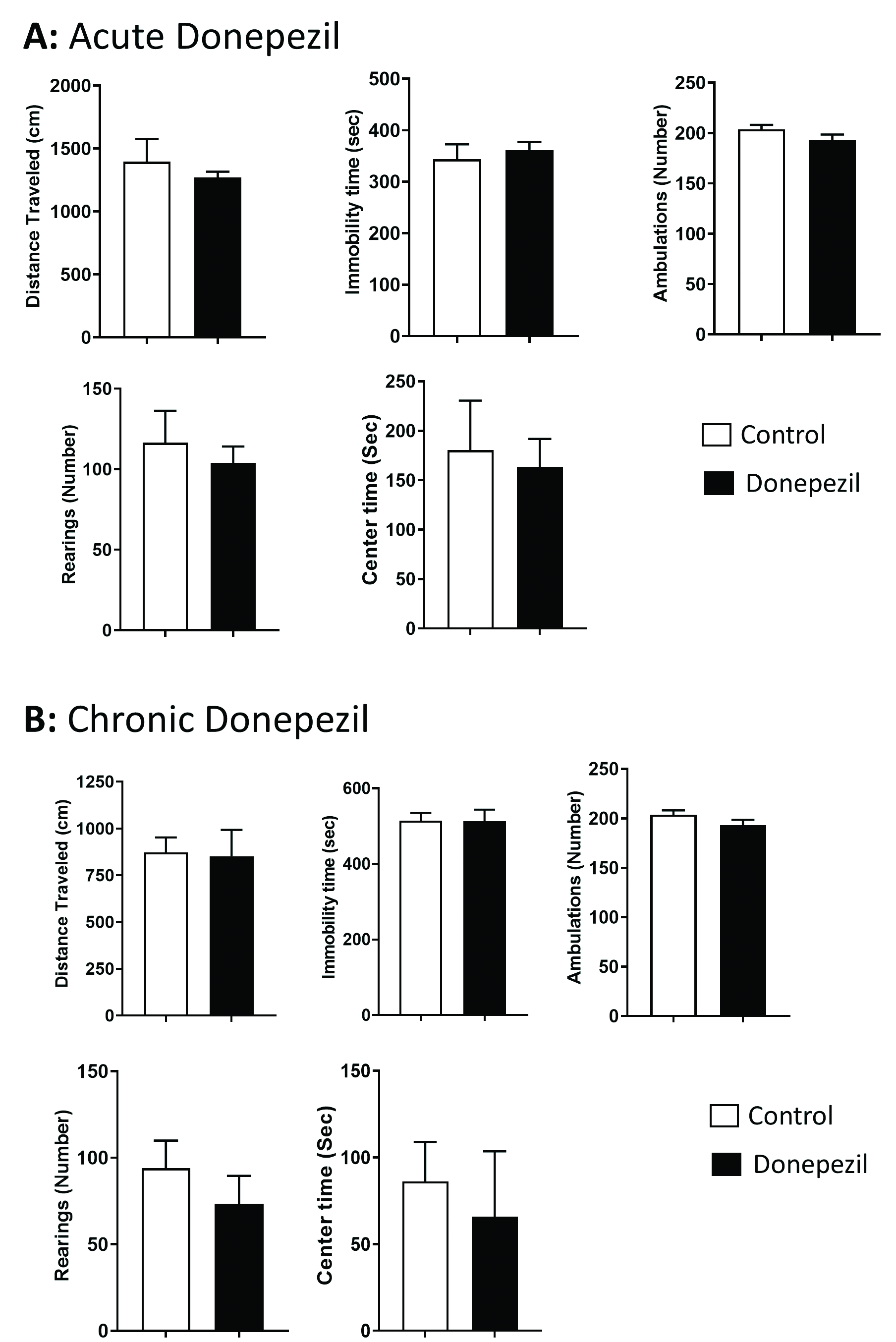

Supplement: Supplementary file 8 — Supplementary Figure 7 [file 41398_2022_1869_MOESM8_ESM.tif]
